# An unusual presentation of tracheobronchial tuberculosis mimicking airway tumor: a case report and literature review

**DOI:** 10.3389/fmed.2026.1918544

**Published:** 2026-09-08

**Authors:** Fei Yuan, Chiqing Ying, Zhengming Huang, Jiaojiao Yang, Angcheng Li

**Affiliations:** 1 Department of Respiratory and Critical Care Medicine, Jinhua Municipal Central Hospital, Jinhua, Zhejiang, China; 2 Department of Respiratory and Critical Care Medicine, Zhejiang Jinhua Guangfu Hospital, Jinhua, Zhejiang, China; 3 Department of Pathology, Jinhua Municipal Central Hospital, Jinhua, Zhejiang, China

**Keywords:** airway tumor, anti-tuberculosis therapy, bronchoscopy, early diagnosis, tracheobronchial stenosis, tracheobronchial tuberculosis

## Abstract

Tracheobronchial tuberculosis is a special type of pulmonary tuberculosis that poses significant diagnostic challenges due to its nonspecific clinical and radiological manifestations. The tumorous subtype of tracheobronchial tuberculosis is particularly rare and carries a variable clinical course with risk of airway stenosis. A 21-year-old male presented with acute-onset cough, chest tightness, and fever. Chest CT revealed a large obstructive endobronchial mass in the left main bronchus and left upper lobe bronchus, initially misdiagnosed as an airway tumor. Given the severe airway obstruction, the patient underwent bronchoscopic resection of the endobronchial mass for diagnosis and symptom relief. Histopathological analysis of the resected mass and molecular testing of bronchoalveolar lavage fluid confirmed tumorous tracheobronchial tuberculosis complicated with infiltrative pulmonary tuberculosis. The patient received individualized anti-tuberculosis therapy tailored to bronchoscopic findings, and follow-up chest CT showed marked lesion regression without complications such as bronchial stenosis. Tumorous tracheobronchial tuberculosis is an unusual, easily misdiagnosed subtype with a potentially severe prognosis, and it must be carefully considered in the differential diagnosis of endobronchial masses. Timely diagnosis and early initiation of anti-tuberculosis therapy are critical for achieving uncomplicated healing, as demonstrated by this patient's favorable outcome.

## Introduction

Tuberculosis (TB) remains a major global public health challenge and is one of the leading causes of death from infectious diseases. According to the Global Tuberculosis Report 2025 of the World Health Organization (WHO), an estimated 10.7 million people fell ill with TB in 2024, with a case fatality rate of 11.5% (1). Tracheobronchial tuberculosis (TBTB), formerly known as endobronchial tuberculosis, is a special type of pulmonary tuberculosis characterized by *Mycobacterium tuberculosis* (MTB) infection involving the mucosa, submucosa, and adventitia of the tracheobronchial tree (2). Due to its atypical clinical presentation and nonspecific radiological findings, TBTB often mimics other conditions, such as malignancy (3, 4), bronchial asthma (5), and airway foreign body aspiration (6). Delayed diagnosis can lead to severe complications, including tracheobronchial stenosis, post-obstructive pneumonia, bronchiectasis, and irreversible pulmonary parenchymal injury. We report a case of tumorous TBTB presenting as a large obstructive endobronchial mass, initially misdiagnosed as an airway tumor. This case highlights the importance of including tuberculosis in the differential diagnosis of such masses, and illustrates a practical, stage-specific airway management strategy that includes diagnostic recanalization and targeted post-diagnosis anti-tuberculosis treatment and may inform clinical decision-making for similar cases.

## Case presentation

**Figure 1:**
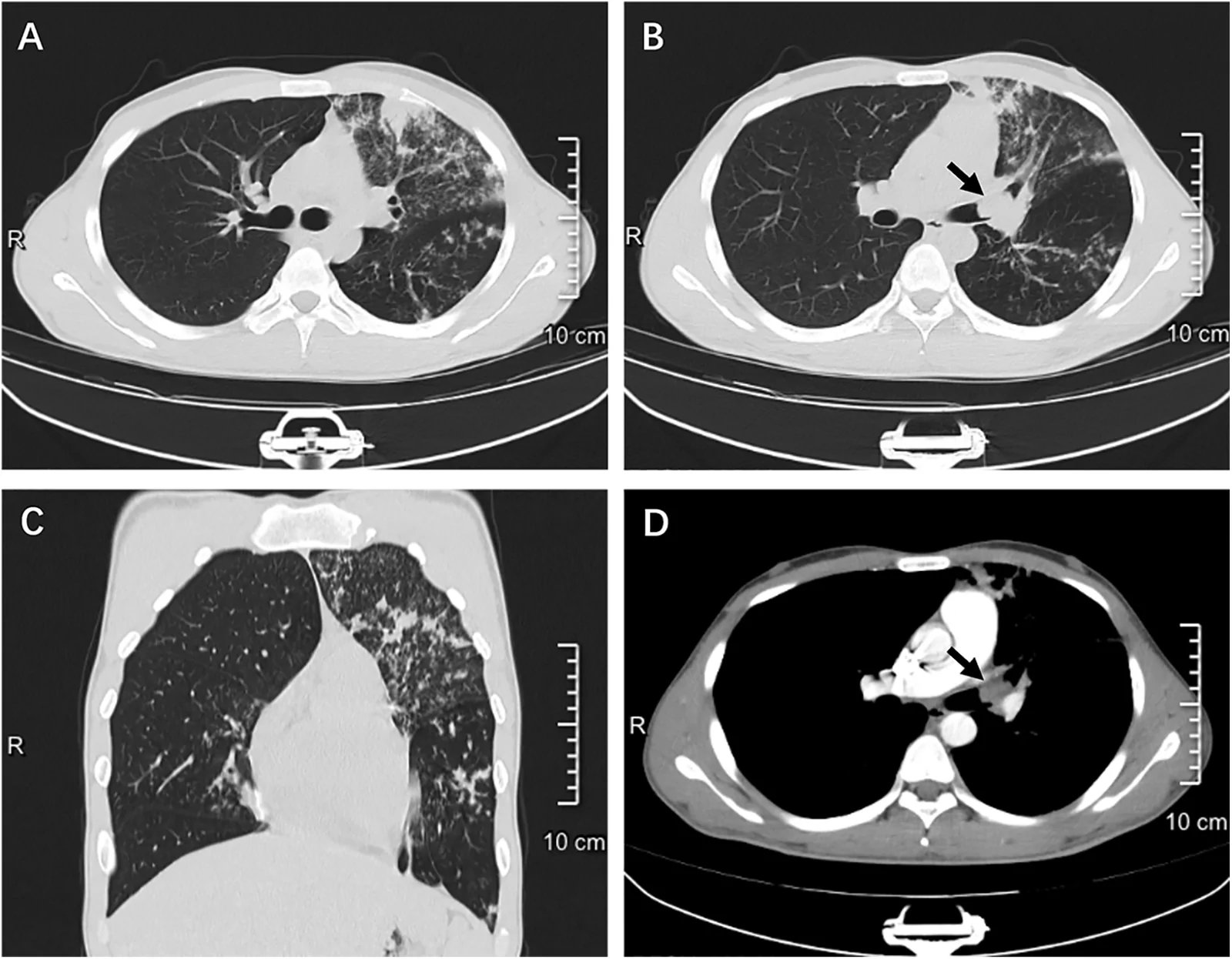
Chest CT demonstrates scattered patchy and speckled high-density opacities with ill-defined margins, predominantly in the left lung, along with a soft tissue attenuation lesion (black arrow) within the left bronchus. **(A,B)** Lung window images; **(C)** Coronal view image; **(D)** Mediastinal window image of contrast-enhanced CT.

A 21-year-old Han Chinese male presented to our Respiratory and Critical Care Medicine outpatient clinic with a one-week history of nonproductive cough, followed by acute chest distress and fever for half a day. As a hotel staff, he was previously healthy, denied any history of smoking, foreign body aspiration, or family history of tumors, and had no contact with anyone known or suspected to have TB. The cough initially developed after overexertion, without fever, chest distress, hemoptysis, weight loss, or night sweats, and the patient did not seek medical care until the onset of chest distress and fever. However, the exact temperature at that time was not measured. Chest CT performed in the outpatient clinic revealed a soft tissue attenuation lesion within the left bronchus, accompanied by scattered patchy and speckled high-density opacities predominantly in the left lung (Figures 1A–C). Therefore, the patient was admitted for further evaluation and management.

Upon admission, vital signs were stable: temperature 37.7 °C; respiratory rate 18 breaths/min; heart rate 83 beats/min; blood pressure 118/88 mmHg. Pulse oxygen saturation was 95% on room air. Physical examination revealed a symmetrical thorax without deformity, diminished breath sounds in the left upper lung field, and no prominent crackles in either lung. Cardiac and abdominal examinations showed no significant abnormalities. Given the acute presentation and imaging findings, community-acquired pneumonia was initially suspected, though the mass-like lesion in the left bronchus was also noted. Due to a documented allergy to azithromycin and cephalosporins, empirical intravenous omadacycline (100 mg daily, with a double initial dose) was initiated alongside symptomatic therapies.

Laboratory investigations showed an elevated C-reactive protein (34.99 mg/L) and a normal white blood cell count (8.8 × 10⁹/L) with a neutrophil predominance (75.5%). Myocardial enzyme spectrum, cardiac troponin I, and B-type natriuretic peptide were within reference ranges. Among tumor markers, serum neuron-specific enolase was mildly elevated (22.60 ng/mL), while carcinoembryonic antigen, squamous cell carcinoma antigen, pro-gastrin-releasing peptide, and cytokeratin 19 fragment were within normal limits. HIV serology was negative. No abnormalities were detected in electrocardiography and echocardiography. A subsequent contrast-enhanced chest CT identified a 21 × 13 mm soft tissue attenuation lesion obstructing the left main bronchus (LMB) and the left upper lobe bronchus (LULB) (Figure 1D), raising high suspicion for a tumor.

**Figure 2:**
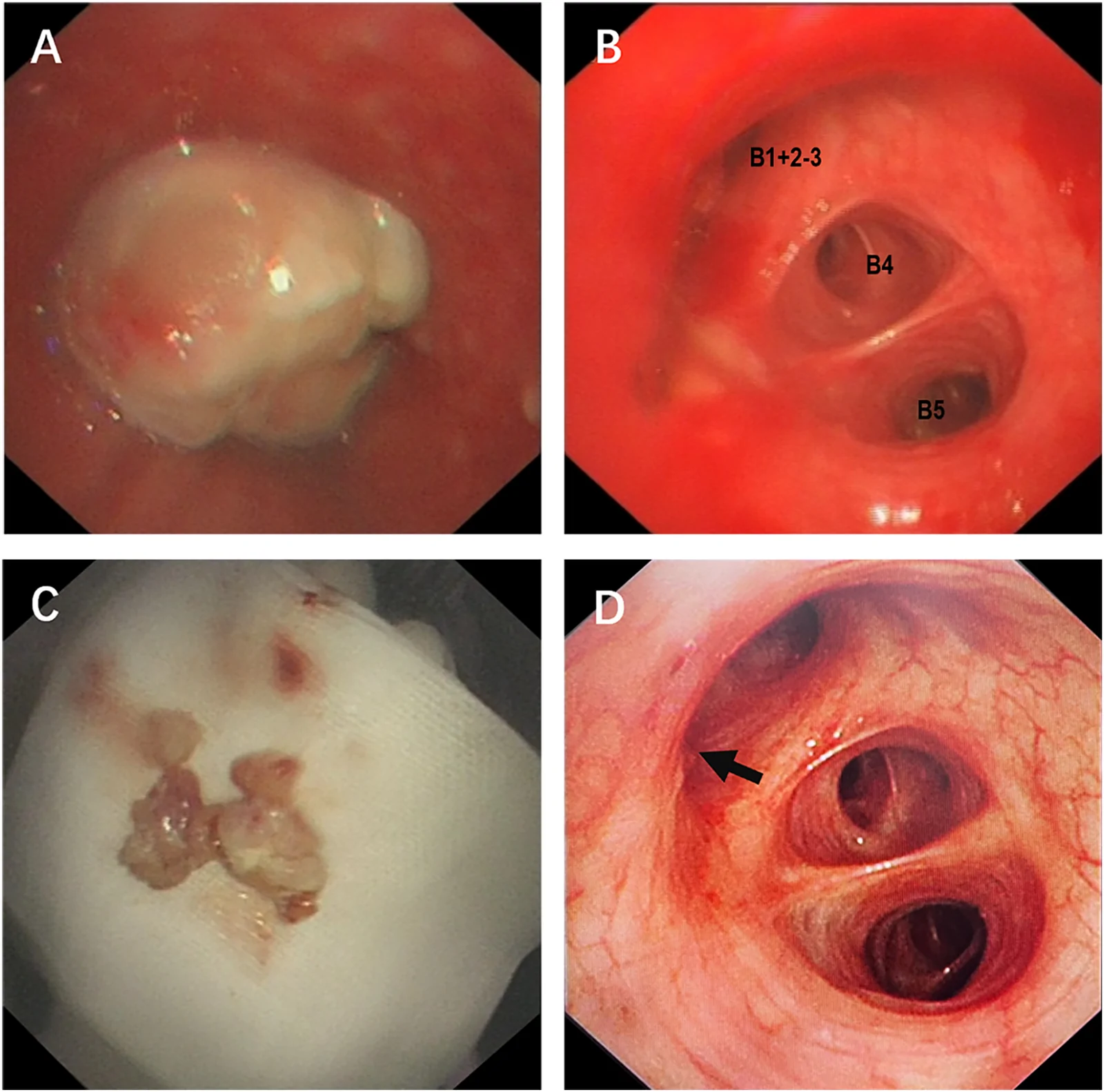
Bronchoscopic and gross pathological findings. **(A)** Initial bronchoscopic view of the LMB, showing near-complete obstruction of its lower segment by an endobronchial neoplasm without caseous surface material. **(B)** View of LULB after neoplasm resection, showing the lesion originating from the proper segmental bronchus (B1 + 2-3: proper segmental bronchus of LULB; B4: superior lingular segment; B5: inferior lingular segment). **(C)** Gross pathological specimen of the resected endobronchial mass. **(D)** Bronchoscopic view during the second intrabronchial instillation, revealing a small nodular lesion (black arrow) in the left upper lobe with intact, smooth overlying mucosa.

To clarify the nature of the bronchial lesion identified on CT, bronchoscopy was performed, which revealed a mass-like lesion causing near-complete obstruction of the lower segment of the LMB, with no caseous material on its surface. High-frequency electrosurgical snare resection of the lesion then followed, during which minimal bleeding was observed and promptly controlled, with no procedural complications. After resection, the lesion was found to originate from the proper segmental bronchus of LULB (Figures 2A–C). Diagnostic bronchoalveolar lavage (BAL) was concurrently performed.

**Figure 3:**
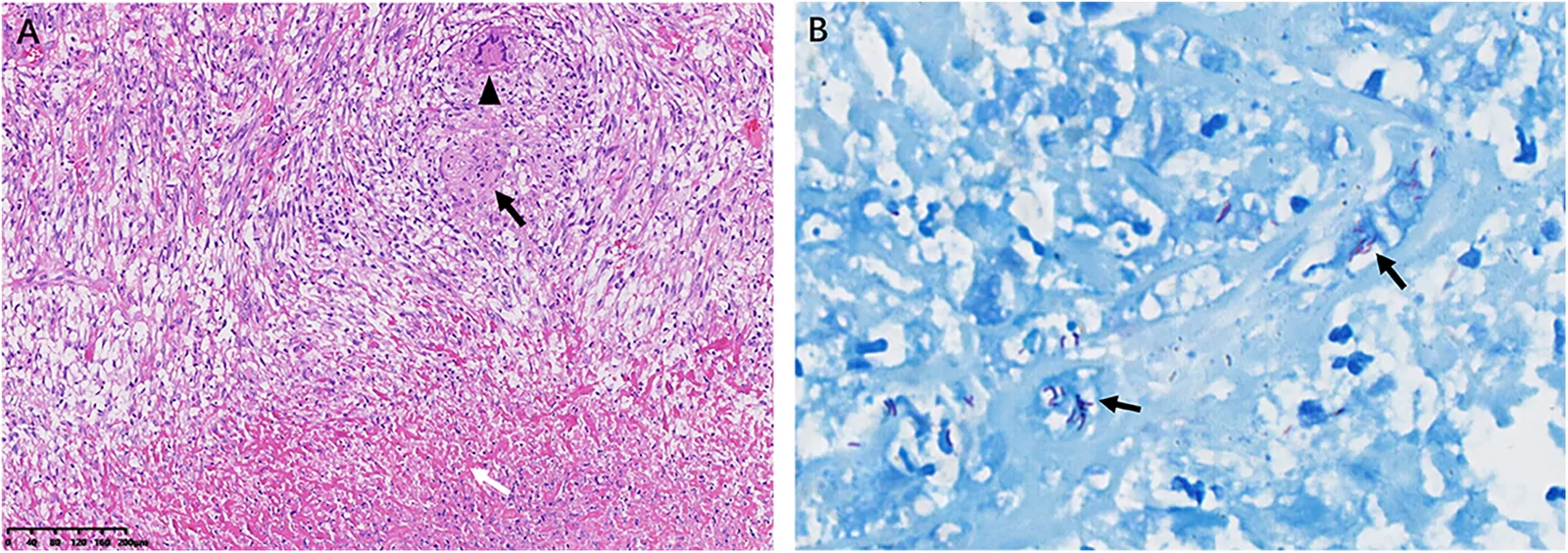
Histopathological and microbiological examination of the resected specimen. **(A)** Histopathological section (hematoxylin and eosin staining; original magnification  × 100) reveals granulomatous inflammation composed of Langhans giant cells (triangle), epithelioid macrophages (black arrow), lymphocytes, and fibroblasts, with necrosis (white arrow). **(B)** Ziehl-Neelsen acid-fast staining (original magnification   ×  400) demonstrates red, thin, rod-like mycobacteria (black arrow).

Acid-fast bacilli were detected in both BAL fluid and post-bronchoscopy sputum. Molecular testing of BAL fluid confirmed MTB via Xpert MTB/RIF (with no rifampicin resistance detected) and targeted next-generation sequencing (tNGS), establishing a diagnosis of pulmonary tuberculosis. The patient was referred to a tuberculosis specialty hospital for anti-tuberculosis treatment, and our department followed up while awaiting histopathological results of the resected bronchoscopic specimen. One week after bronchoscopy, histopathology of the resected specimen revealed diffuse granulomatous inflammation with focal caseous necrosis and no malignant cells; Ziehl-Neelsen staining confirmed acid-fast bacilli (Figure 3), which together provided definitive histomicrobiological diagnostic evidence.

**Figure 4:**
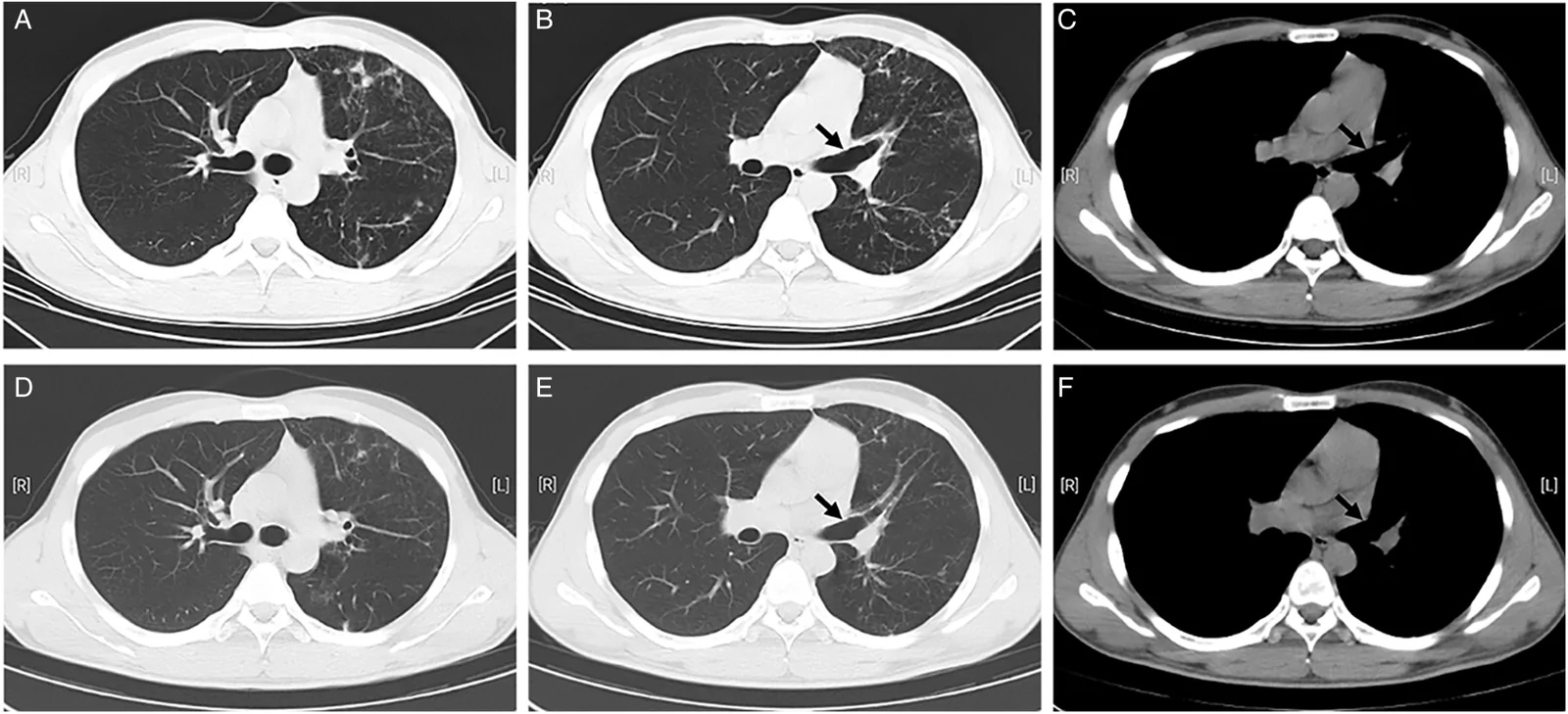
Follow-up chest CT images during anti-tuberculosis therapy reveal progressive radiographic improvement, characterized by the gradual resolution of bilateral pulmonary lesions and sustained patency of the LMB and the LULB. **(A–C)** Images obtained at approximately 2.5 months post-treatment. **(D–F)** Images obtained at 6 months post-treatment. Black arrows in B, C, E, and F indicate the previous lesion site.

Further evaluation confirmed the diagnosis of TBTB complicated with infiltrative pulmonary tuberculosis, and the patient received individualized combination anti-tuberculosis therapy: the 2-month intensive-phase standard HRZE regimen (isoniazid [H] 300 mg, rifampicin [R] 600 mg, ethambutol [E] 750 mg — all oral, once daily; pyrazinamide [Z] 500 mg three times daily) plus a 1-month course of nebulized HR therapy (isoniazid 100 mg and rifampicin 225 mg, once daily via nebulization). After completion of the intensive phase, the patient transitioned to the consolidation-phase with oral HR, which was planned for a total duration of 10 months. Furthermore, two targeted bronchoscopic instillations of isoniazid (100 mg per session) were delivered directly into the left upper lobe bronchus at a 4-week interval. During the second bronchoscopic instillation, a small nodular lesion with intact, uniformly smooth mucosa was identified in the left upper lobe (Figure 2D). Follow-up chest CT examinations at approximately 2.5 months and 6 months post-treatment showed progressive radiographic improvement, sustained bronchial patency, and no evidence of stenosis (Figure 4). After more than six months of therapy, the patient achieved sputum smear conversion and reported complete resolution of symptoms, with no limitations in daily life, work, or physical activity. As of manuscript preparation, the patient remains on consolidation-phase treatment (oral HR) for recurrence prevention.

## Discussion

TBTB is defined as MTB infection of the tracheobronchial tree, confirmed microbiologically or histopathologically, with or without pulmonary parenchymal involvement (7). The reported incidence of TBTB in patients with active pulmonary tuberculosis is highly variable, ranging from 5.88% to 54.3% in prospective studies (8–11). The pathogenesis of TBTB remains incompletely understood. Proposed mechanisms include: (a) direct extension from an adjacent pulmonary parenchymal lesion; (b) implantation of organisms from the infected sputum; (c) hematogenous spread; (d) adjacent tuberculous lymph node erosion into a bronchus; and (e) extension to the peribronchial region via lymphatic drainage (8, 12, 13). In this case, the patient had TBTB complicated with pulmonary parenchymal involvement, and contrast-enhanced chest CT showed no significant lymphadenopathy in adjacent hilar, mediastinal, or peribronchial regions. This suggests that the most likely infection mechanisms are direct extension from an adjacent pulmonary parenchymal lesion or endobronchial implantation via infected sputum.

TBTB is a special type of pulmonary tuberculosis. Pulmonary tuberculosis typically has an insidious onset, and most patients follow a subacute or chronic clinical course with symptoms (e.g., cough, expectoration, chest pain, hemoptysis, dyspnea, fever, weight loss, and fatigue) persisting for weeks to months. Cough is the most common symptom in patients with TBTB (10). TBTB shows a marked female predominance in the second and third decades of life (8, 14). However, a prospective study has revealed a gender-specific bimodal age distribution in TBTB incidence, with distinct peaks among females in their 20s and those aged ≥70 years (11). Female gender and symptom duration ≥4 weeks have been identified as the main predictors of pulmonary tuberculosis complicated by TBTB in previous studies (9, 10). Contrary to these typical features, our 21-year-old male patient presented with an acute onset of symptoms, lacking the classic clinical indicator. Radiologically, the patient had a bronchial soft-tissue lesion and scattered ill-defined patchy and speckled high-density opacities predominantly in the left lung. Therefore, the patient was initially misdiagnosed as an airway tumor complicated with acute bacterial pneumonia based on these clinical and radiographic findings. However, other infectious etiologies such as parenchymal pulmonary tuberculosis and fungal infections required exclusion.

Bronchoscopy is the most reliable method for diagnosing TBTB, which not only provides biological samples for etiological identification but also enables evaluation of endobronchial lesions (15). Chung et al. (8) classified TBTB into seven endobronchial subtypes based on bronchoscopic morphology: actively caseating, edematous-hyperemic, fibrostenotic, tumorous, granular, ulcerative, and nonspecific bronchitic. This classification, closely linked to disease progression, is widely recognized. Additionally, drawing on the main morphological changes and interventional treatment responses observed during bronchoscopy, Chinese scholars further classified TBTB into nine subtypes in the 2025 guidelines for TBTB issued by the Chinese Society of Tuberculosis under the Chinese Medical Association: inflammatory infiltration, ulcer necrosis, granulation tissue proliferation, lymph node fistula, wall fistula, wall softening, scar stenosis, lumen occlusion, and repeated retraction (2). Among the subtypes in Chung's classification of TBTB, the tumorous type—approximately corresponding to the granulation tissue proliferation type in the Chinese guideline—is characterized by an endobronchial mass covered with caseous material, which can cause near-total bronchial lumen occlusion. In previous studies, the actively caseating and edematous-hyperemic subtypes were the most common TBTB subtypes, while tumorous TBTB was a relatively rare subtype (8–10).

As a relatively rare subtype, tumorous TBTB is frequently misdiagnosed as malignancy due to overlapping bronchoscopic and radiological features. Among previously published case reports (3, 16–18), most such cases presented as endobronchial masses on bronchoscopy, with chest CT findings mimicking lung cancer (including endobronchial lesions, atelectasis, hilar or mediastinal lymphadenopathy, and parenchymal soft-tissue opacities). In our case, the patient had a large obstructive endobronchial mass—a size unreported in prior tumorous TBTB cases (3, 16–18). Notably, unlike the literature-described lung cancer-overlapping CT features (such as atelectasis, lymphadenopathy, parenchymal opacities), our patient's CT lacked these signs. Thus, we initially suspected a benign airway tumor, which needed to be differentiated from common benign lesions such as lipoma, papilloma, leiomyoma, and hamartoma (19)—these were prioritized based on the patient's non-specific clinical manifestations and imaging features. However, histopathology confirmed a tuberculous granuloma, with molecular testing further verified tuberculosis. The large granuloma likely predated acute symptoms, this delayed presentation may be attributed to the disease's insidious onset, non-specific symptoms, decreased symptom perception, or underlying immune dysfunction. Additionally, enhanced MTB virulence and sudden immune decline may have driven rapid lesion progression.

Tracheobronchial stenosis is the most common and severe complication of TBTB, affecting over 90% of patients (20, 21) and leading to obstructive pneumonia and exertional dyspnea (22), potentially requiring repeated bronchoscopic interventions or even surgery. Eradicating MTB and preventing tracheobronchial stenosis are the main goals of TBTB treatment. TBTB treatment is similar to that of pulmonary tuberculosis, with its course and prognosis mainly tied to pretreatment lesion degree, extent, and duration (23). Notably, prognostic outcomes vary significantly among different subtypes of TBTB, which is critical for clinical decision-making. Chung and Lee's studies have found that the evolution of lesions in tumorous TBTB during treatment is highly complex, and its prognosis is most grave and unpredictable (8, 24). Given our patient is diagnosed with the tumorous subtype, close follow-up monitoring for potential stenosis is required. Therefore, early diagnosis and timely, standardized anti-tuberculosis treatment are essential to prevent such complications in TBTB management.

Systemic anti-tuberculosis therapy is the cornerstone of TBTB treatment. Additionally, bronchoscopic intervention for TBTB requires individualized strategies based on bronchoscopic morphological lesion classification, with primary techniques including cryotherapy, balloon dilation, thermal ablation (laser, electrocautery, argon plasma coagulation), and stenting (2). In this case, our patient underwent preliminary diagnostic-therapeutic bronchoscopic electrosurgical snare resection of the endobronchial mass in the lower segment of the LMB prior to definitive diagnosis. As tuberculosis was not initially suspected, the patient presented with nearly complete occlusion of the lower segment of the LMB, which significantly impaired his respiratory function. We did not consider a simple forceps biopsy appropriate, as it would only yield diagnostic tissue without addressing the airway obstruction. Thus, this procedure was performed for both tissue diagnosis and restoration of airway patency.

Following confirmation of the diagnosis, the patient commenced systemic anti-tuberculosis therapy with the standard HRZE regimen. However, it has been reported that bronchial stenosis may develop in spite of effective oral anti-tuberculosis therapy (25), which may be attributed to the difficulty of systemic anti-tuberculosis therapy in achieving locally effective drug concentrations at bronchial lesions. Local anti-tuberculosis therapies, such as nebulized inhalation and bronchoscopic intraluminal drug instillation, address this pharmacokinetic limitation. To date, no internationally standardized regimen exists for local anti-tuberculosis treatment. As a young male patient deeply concerned about potential complications (e.g., bronchial stenosis) affecting his quality of life and daily functioning, he actively expressed a strong preference for local anti-tuberculosis therapies, aiming to minimize long-term sequelae. Taking into account his participation in treatment decision-making and following the Chinese guidelines for TBTB (26) and the expert consensus on clinical use of anti-tuberculosis drugs (27), we administered daily nebulized inhalation of isoniazid and rifampicin, along with bronchoscopy-guided intrabronchial instillation of isoniazid into the involved bronchial segment. However, given the lack of internationally standardized regimens and the individualized nature of this decision, the generalizability of this local treatment strategy requires further validation through large-scale clinical studies. Systemic corticosteroid combined with anti-tuberculosis therapy can rapidly relieve TB symptoms, but their role in preventing TBTB-related bronchial stenosis remains controversial. Some studies found no clinically meaningful improvement in TBTB patient outcomes with corticosteroids (28, 29). Thus, the patient received anti-tuberculosis therapy alone.

Due to the unpredictable prognosis of tumorous TBTB and risk of bronchial stenosis, long-term follow-up is recommended (24). Bronchoscopy is essential for follow-up of this subtype, enabling direct visualization of mucosal healing, residual granuloma, and early fibrostenosis, which are subtle but critical changes for timely intervention often missed by imaging alone. While Lee and Chung (24) demonstrated that chest CT can partially compensate for bronchoscopy's inability to fully evaluate extraluminal or distant lesions (including bronchial involvement length, luminal patency, and peribronchial thickness), a retrospective study suggested that CT may replace bronchoscopy for evaluating therapeutic response and complications (30). However, this finding should be interpreted with caution: CT lacks the spatial resolution to detect the early mucosal or submucosal changes that bronchoscopy identifies with high sensitivity. In this case, the patient declined follow-up bronchoscopy after the initial diagnostic and therapeutic procedure. As an alternative, the patient opted for serial follow-up CT imaging, which has demonstrated stable disease without radiological signs of bronchial stenosis to date. While CT confirmed macroscopic stability, a key limitation of this approach is its inability to assess mucosal microstructural healing or residual granulomas, which are factors that may precede clinically overt stenosis. Thus, the decision to forgo follow-up bronchoscopy introduces uncertainty: we cannot rule out subclinical mucosal inflammation or early fibrostenosis that may progress silently without clinical symptoms. Accordingly, future studies should explore strategies to balance patient preferences with the need for gold-standard monitoring.

## Conclusion

TBTB presenting as an endobronchial mass is a well-recognized but frequently overlooked mimic of airway tumor. Clinicians must maintain a high index of suspicion and include TBTB in the differential diagnosis of endobronchial masses particularly in young patients without classic constitutional symptoms. Prompt diagnostic intervention and individualized systemic and local therapies are vital to preventing irreversible airway stenosis and achieving a complete clinical cure. Given the unpredictable prognosis of tumorous TBTB and the risk of silent progression of subclinical mucosal changes, future practice should emphasize that follow-up CT imaging (for macroscopic stability) and bronchoscopy (for early mucosal changes) are both essential for comprehensive monitoring.

## Data Availability

The original contributions presented in the study are included in the article/Supplementary Material, further inquiries can be directed to the corresponding author.
