# Supplementary material for: An unusual presentation of tracheobronchial tuberculosis mimicking airway tumor: a case report and literature review
Source: Front Med (Lausanne). 2026 Sep 8;13:1918544. doi: 10.3389/fmed.2026.1918544 (PMC13593766; doi:10.3389/fmed.2026.1918544)
Supplement: Supplementary file 1 [file Image1.pdf]

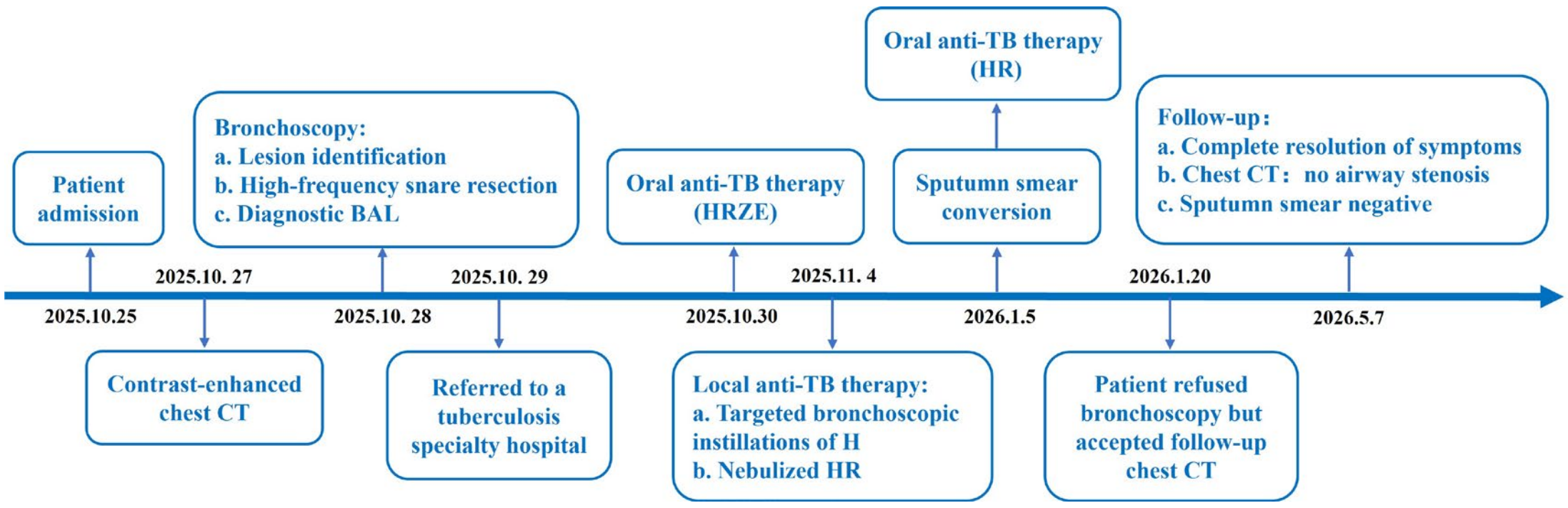

Supplementary Figure 1 Timeline of the patient's care episode. H: isoniazid; R: rifampicin; Z: pyrazinamide; E: ethambutol; BAL: bronchoalveolar lavage.
